# Rho/ROCK Signal Cascade Mediates Asymmetric Dimethylarginine-Induced Vascular Smooth Muscle Cells Migration and Phenotype Change

**DOI:** 10.1155/2014/683707

**Published:** 2014-07-09

**Authors:** Yi-ming Zhou, Xi Lan, Han-bin Guo, Yan Zhang, Li Ma, Jian-biao Cao

**Affiliations:** ^1^Department of Liver Disease, The Military General Hospital of Beijing PLA, Beijing 100072, China; ^2^Department of Anesthesiology and Critical Care Medicine, School of Medicine, Johns Hopkins University, Baltimore, MD 21205, USA; ^3^Department of Clinical Laboratory, The Military General Hospital of Beijing PLA, Beijing 100072, China

## Abstract

Asymmetric dimethylarginine (ADMA) induces vascular smooth muscle cells (VSMCs) migration. VSMC phenotype change is a prerequisite of migration. RhoA and Rho-kinase (ROCK) mediate migration of VSMCs. We hypothesize that ADMA induces VSMC migration via the activation of Rho/ROCK signal pathway and due to VSMCs phenotype change. ADMA activates Rho/ROCK signal pathway that interpreted by the elevation of RhoA activity and phosphorylation level of a ROCK substrate. Pretreatment with ROCK inhibitor, Y27632 completely reverses the induction of ADMA on ROCK and in turn inhibits ADMA-induced VSMCs migration. When the Rho/ROCK signal pathway has been blocked by pretreatment with Y27632, the induction of ERK signal pathway by ADMA is completely abrogated. Elimination of ADMA via overexpression of dimethylarginine dimethylaminohydrolase 2 (DDAH2) and L-arginine both blocks the effects of ADMA on the activation of Rho/ROCK and extra cellular signal-regulated kinase (ERK) in VSMCs. The expression of differentiated phenotype relative proteins was reduced and the actin cytoskeleton was disassembled by ADMA, which were blocked by Y27632, further interpreting that ADMA inducing VSMCs migration via Rho/ROCK signal pathway is due to its effect on the VSMCs phenotype change. Our present study may help to provide novel insights into the therapy and prevention of atherosclerosis.

## 1. Introduction

Atherosclerosis and its complications, including myocardial infarction, stroke, and peripheral vascular disease, remain a major cause of death in industrialized countries [1, 2]. VSMCs migration plays a critical role in the initiation and progress of intimal thickening in atherosclerotic lesions [3, 4].

VSMCs phenotype change is a prerequisite of migration. In a mature blood vessel, VSMCs exhibit differentiated phenotype characteristics, such as the expression of contractile markers specific to smooth muscle, the assembly of actin cytoskeleton into parallel, and the elongated stress fibres. However, VSMCs retain remarkable plasticity and can undergo rather profound and reversible changes in phenotype in response to changes in local environmental cues [5]. They demonstrate an increased rate of proliferation and migration, exhibit a decrease in expression of SM-specific contractile markers, show disassembly and aggregation of actin fibres around the perinuclear region, and change from a spindle-like to a polygonal shape.

Many different intracellular signaling molecules have been implicated in VSMCs migration, including the small G protein RhoA and its effector ROCK, mitogen-activated protein kinase (MAPK) cascades [6, 7]. Recently, asymmetric dimethylarginine (ADMA), an endogenous inhibitor of nitric oxide synthase (NOS) and a newly found cardiovascular risk factor, has been reported to induce VSMCs migration via MAPK cascades [8]. However, whether ADMA induces VSMCs migration via Rho/ROCK cell signaling transduction way and whether ADMA induces VSMCs migration via phenotype change are still unknown.

Based upon previous observations, we hypothesized that ADMA may induce VSMCs phenotype change from being differentiated into the dedifferentiated state via the activation of Rho/ROCK, leading to VSMCs migration.

Investigations to identify the relation between ADMA and VSMCs migration may help to provide novel insights into the therapy and prevention of atherosclerosis.

## 2. Materials and Methods

### 2.1. Antibodies and Major Reagents

ADMA, symmetric dimethylarginine (SDMA), L-arginine, and Y27632 were purchased from Sigma (Oakville, ON, USA). Rhotekin-RBD GST beads were purchased from Cytoskeleton (Denver, CO, USA). 4,6-diamidino-2-phenylindole (DAPI) and propidium iodide (PI) were all purchased from BIOMOL (Waidmannstraße, Hamburg, Germany). Alexa Fluor 568 phalloidin was purchased from Invitrogen (Camarillo, CA, USA). FuGENE HD transfection reagent was purchased from Roche (Basel, Switzerland). Antibodies were purchased from the following sources. Thr^853^-phosphorylated myosin phosphatase target subunit-1 (MYPT-1), MYPT-1, Thr^202/204^-phosphorylated-p44/42 MAPK (Erk1/2), and p44/42 MAPK were from Cell Signal Technology (Danvers, MA, USA). Mouse monoclonal antibody to rat smooth muscle alpha actin (*α*-actin) was from Thermo Scientific (Rockford, IL, USA). Monoclonal antibody to rat Rho was purchased from Millipore (Oak, CA, USA). Isotype-matched fluorescein isothiocyanate- (FITC-) conjugated anti-rat IgG1 secondary antibody was purchased from Invitrogen (Camarillo, CA, USA).

### 2.2. Cell Culture

The experimental protocol conforms to the Guide for the Care and Use of Laboratory Animals published by the US National Institutes of Health (NIH Publication Number 85-23, revised 1996) and is approved by the Beijing General Hospital of Beijing Military Command. Culture of VSMCs derived from the carotid arteries of male Sprague-Dawley rats was done as previously described [8]. Only VSMCs from passage three to five were used for the experiments. To distinguish whether the effects of ADMA on VSMCs migration are NO dependent, we used DMEM (without L-arginine) from Gibco (Auckland, New Zealand) as the growth medium.

### 2.3. DDAH2 Overexpression

ADMA is hydrolyzed to L-citrulline and dimethylamine by dimethylarginine dimethylaminohydrolase 2 (DDAH2) in the cardiovascular system. The strategies of DDAH2 overexpression were used to modulate the level of ADMA. The human DDAH2 overexpression plasmid vector pAV-CMV-GFP-DDAH2 or empty vector pAV-CMV-GFP (friendly gifted by Prof. L. Sun, Institute of Materia Medica, Chinese academy of medical sciences) was transiently transfected into rat VSMCs. Transfections were performed using FuGENE HD according to the manufacturer's guidelines. All transfections were carried out in triplicate, and each experiment was reproduced four to six times. The transfection efficiency by these methods was approximately 60% to 70%.

### 2.4. VSMCs Migration Assay

Cell migration was performed using the wound-healing assay as previously described [8]. Confluent VSMCs (starved for 48 h in FCS-free DMEM without L-arginine) were treated with ADMA or without ADMA at the concentrations of 10 *μ*M for 24 h. The migration/growth medium was DMEM without L-arginine containing 5 mM hydroxyurea to prevent VSMCs proliferation. Four different fields of migration were photographed with a video camera system using Image Pro Plus 5.1 software. Samples were run in triplicate in three different experiments.

### 2.5. VSMCs Proliferation Assay

BrdU incorporation assay was used to detect VSMCs proliferating ability. Briefly, VSMCs were seeded at 50% confluence in Costar 96-well plates. Cells were starved in FCS-free medium for the next 48 h and pretreated with or without Y27632 for 24 h at the concentration of 10 *μ*M and then treated with ADMA (10 *μ*M) for 48 h or not. BrdU (10 *μ*M) was added to the medium 5 h before harvesting. Cells were trypsinized, fixed in ice-cold ethanol (70%), and treated with HCl (2 M) and subsequently with sodium borate buffer (0.1 M, pH 8.5). After incubation with mouse anti-BrdU antibody and Isotype-matched fluorescein isothiocyanate- (FITC-) conjugated anti-rat IgG1 secondary antibody, DAPI was employed to detect nuclei. Labeled cells were examined under a Zeiss confocal microscope, and images were obtained with the laser scanning confocal microscope LEICA TCS SP2 (Leica, Wetzlar, Germany).

### 2.6. Rho Activity Assay

VSMCs were lysed with an ice-cold cell lysis buffer. Cell lysates were incubated with GST-fused Rho-binding domain of Rhotekin which were bound to glutathione-Sepharose beads for one hour, and bound proteins were immunoblotted with anti-RhoA antibody [9].

### 2.7. Western Blotting Assay

The cellular lysates and western blotting analysis were performed as previously mentioned [10]. The proteins were separated by 12% SDS-PAGE and transferred onto nitrocellulose membrane (Millipore, Bedford, MA, USA). The polyvinylidene fluoride membranes were blocked with 5% bovine serum albumin. After washing, the blots were then probed with specific primary antibodies and second antibodies. Membranes were visualized with a chemiluminescence system (ChemiDoc XRS, Bio-Rad, PA, USA). The bands on the films were quantified by Quantity One software (Bio-Rad, CA, USA).

### 2.8. Immunofluorescence and Confocal Laser Scanning Microscopy

Immunofluorescence staining was used to study F-actin and cell morphology. After fixation with 3.7% paraformaldehyde for 15 min and permeabilization with 0.2% Triton X-100 in phosphate buffer solution for 10 min, the slides were incubated with blocking buffer (3% BSA, 0.1% Tween 20 in Tris buffer solution). Then the slides were incubated with Alexa Fluor 568 phalloidin to detect F-actin, and PI was employed to detect nuclei [11]. Labeled cells were examined under a Zeiss confocal microscope, and images were obtained with the laser scanning confocal microscope LEICA TCS SP2 (Leica, Wetzlar, Germany).

### 2.9. Statistical Analysis

Data are expressed as mean ± SEM. Each experiment was repeated three times, and representative results are shown. The Kolmogorov-Smirnov method was used to test the normal distribution. One-way ANOVA was used to analyze values, followed by Student's *t*-tests to distinguish significant differences. A *P* value < 0.05 was considered significant.

## 3. Results

### 3.1. ADMA Upregulates RhoA Activity

**Figure 1:**
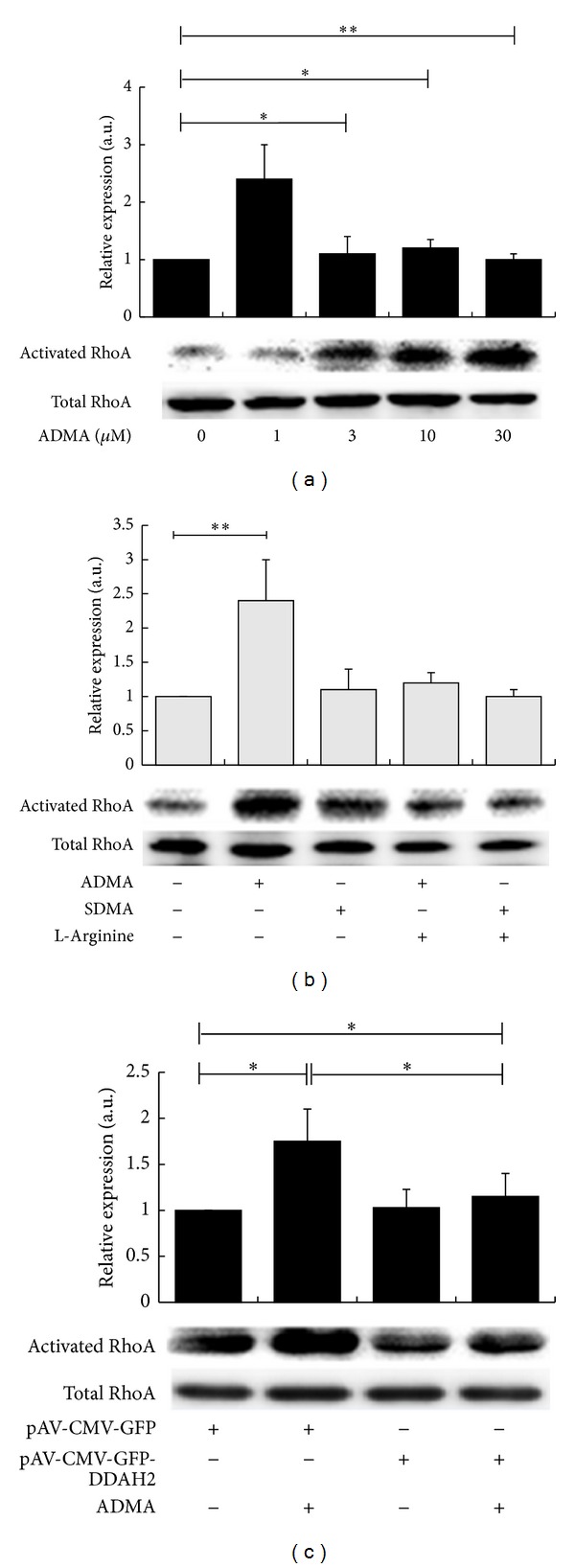
ADMA upregulates RhoA activity. (a) Confluent VSMCs (starved for 48 h in FCS-free DMEM without L-arginine) were treated with ADMA at different concentrations (0-30 *μ*M) for 30 min. (b) VSMCs were pretreated with L-arginine (1 mM) for 2 h and then incubated with ADMA or SDMA at the concentration of 10 *μ*M for 30 min. (c) VSMCs were transfected with the reconstitute human DDAH2 overexpression plasmid vector (pAV-CMV-GFP-DDAH2) or the blank vector pAV-CMV-GFP for 48 h and then incubated with ADMA at the concentration of 10 *μ*M for 30 min. VSMCs were lysed with an ice-cold cell lysis buffer. Cell lysates were incubated with GST-fused Rho-binding domain of rhotekin which were bound to glutathione-Sepharose beads for 1 h, and bound proteins were immunoblotted with anti- RhoA antibody. The graphs represent the ratio of pull-down RhoA to total RhoA of every group to VSMCs without ADMA treatment. Data shown are the mean ± SEM from three independent experiments, *P < 0.05;  **P < 0.01.

The affinity precipitation of RhoA with GST-rhotekin fusion protein indicates the activity of small G protein, RhoA. As shown in Figure 1(a), ADMA activated RhoA in a concentration-dependent manner in the concentration range of 1–30 *μ*M, as assessed by the affinity precipitation of RhoA. Treatment of VSMCs with ADMA at the concentration of 10 *μ*M evidently increased RhoA activity to a 1.9-fold by comparing to VSMCs without ADMA treatment (*P* < 0.05).

To investigate whether the inducing effects on RhoA were ADMA specific, the same concentrations of SDMA, an ADMA analogue, were used as controls. As is shown in Figure 1(b), SDMA did not increase affinity precipitation of RhoA with GST-rhotekin fusion protein. The precursor of NO, L-arginine markedly attenuated RhoA activity induced by ADMA in VSMCs.

To further confirm the role of the DDAH2/ADMA in regulating the activity of RhoA, VSMCs were transfected with a constitutively expression plasmid (pAV-CMV-GFP-DDAH2) and then treated with ADMA at a concentration of 10 *μ*M for 30 min. As is shown in Figure 1(c), overexpression of DDAH2 in VSMCs markedly reduced the affinity precipitation of RhoA to a normal level by comparing to VSMCs infected with the blank vector pAV-CMV-GFP (*P* < 0.05).

### 3.2. ADMA Induces VSMCs Migration and Proliferation via RhoA/ROCK Cell Signaling Transduction Way

**Figure 2:**
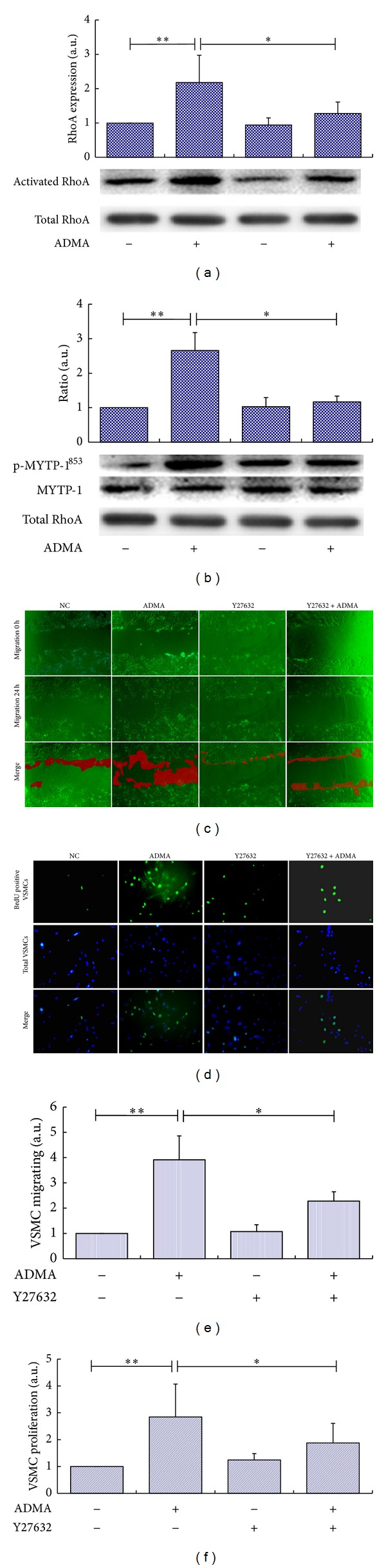
ADMA induces VSMCs migration and proliferation via RhoA/ROCK cell signaling transduction way. (a) The affinity precipitation of RhoA with GST-rhotekin fusion protein. The graphs represent the ratio of pull-down RhoA to total RhoA of every group to VSMCs without ADMA treatment. (b) Phosphorylation level of the MYTP-1 in VSMCs. Confluent VSMCs were pretreated with Y27632 (10 *μ*M) or not and then incubated with ADMA at the concentration of 10 *μ*M for 30 min. (c) The migration ability of VSMCs was assessed by the confluent area of VSMCs in a wound-healing assay. Confluent VSMCs were pretreated with Y27632 or not (10 *μ*M, 2 h) and then incubated with ADMA at the concentration of 10 *μ*M for 24 h. The upper, middle, and bottom images represent the beginning point (no incubation with ADMA), end point (incubation with ADMA for 24 h), and merged images, respectively. The red shadow represents the change in covered area. (e) The migration activity was expressed as the X-folds increase of the change in covered area by comparing to VSMCs without ADMA treatment. (d) The proliferation ability of VSMCs was assessed by the DNA synthesis rate of VSMCs in a BrdU incorporation assay. After incubation with mouse anti-BrdU antibody and Isotype-matched fluorescein isothiocyanate- (FITC-) conjugated anti-rat IgG1 secondary antibody, DAPI was employed to detect nuclei. The upper, middle, and bottom images represent the BrdU positive VSMCs, nucleolus of all VSMCs in an observation field, and merged images, respectively. (f) The proliferation activity was expressed as the X-folds increase of the synthesis rate by comparing to VSMCs without ADMA treatment. Data shown are the mean ± SEM from three independent experiments, *P < 0.05;  **P < 0.01.

Thr^853^-phosphorylation of MYPT-1 represents the activation of ROCK. As shown in Figure 2(a), treatment with ADMA for 30 minutes markedly upregulated the affinity precipitation of RhoA with GST-rhotekin fusion protein (*P* < 0.05) and hence leads to a 2.5-folds increase of the phosphorylation level of MYPT-1 at Thr^853^ in VSMCs (Figure 2(b), *P* < 0.05). Pretreatment of VSMCs with a selective ROCK inhibitor, Y27632 (10 *μ*M, 30 min), reduced the phosphorylation of MYPT-1 to a normal level (*P* < 0.05).

Then, we detected the direct role of Rho/ROCK on the migration and proliferation of VSMCs induced by ADMA. VSMCs pretreated with Y27632 were cultured with ADMA at a concentration of 10 *μ*M. The migration ability of VSMCs was assessed by the confluent area of VSMCs in a wound-healing assay (Figures 2(c) and 2(e)). Treatment of VSMCs with 10 *μ*M ADMA leads to a 3.9-fold increase in VSMCs migration (*P* < 0.01), while the ADMA analogue, SDMA, did not. Y27632 obviously blocked VSMCs migration induced by ADMA (*P* < 0.05).

As a next step, we tested the influence of ADMA on the DNA synthesis (BrdU incorporation) in VSMCs. As is shown in Figures 2(d) and 2(f), pretreatment with ADMA leads to a 2.5-fold increase in DNA synthesis in VSMCs (*P* < 0.01), and Y27632 obviously blocked DNA synthesis in VSMCs induced by ADMA (*P* < 0.05).

### 3.3. ADMA Changes the Expression of Differentiated Markers via the Activation of Rho/ROCK

**Figure 3:**
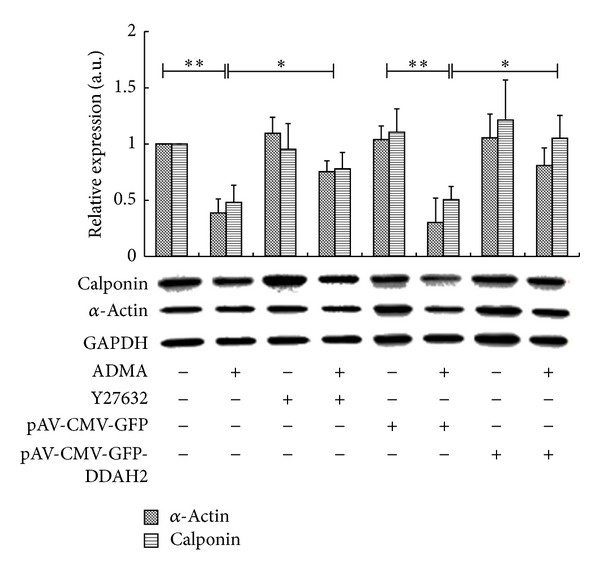
ADMA changes the expression of differentiated markers via the activation of Rho/ROCK. Confluent VSMCs were pretreated with Y27632 (or not) at the concentration of 10 *μ*M or overexpression of pAV-CMV-GFP-DDAH2 (or the blank vector of pAV-CMV-GFP) and then incubated with or without ADMA at the concentration of 10 *μ*M for 12 h. The expression level of differentiated related markers of *α*-actin and calponin was detected by Western Blotting. GAPDH was used as internal control. The graphs represent the relative expression level of every group by comparing to wild type VSMC without ADMA treatment. Data shown are the mean ± SEM from three independent experiments, *P < 0.05;  **P < 0.01.

The change of differentiated marker (such as SM *α*-actin and calponin) expression level in VSMCs is a useful paradigm for analysis of VSMCs phenotypic transition. Treatment of ADMA for 12 h at the concentration of 10 *μ*M significantly reduced the differentiated marker protein expression by assessing the relative expression level of *α*-actin and calponin (Figure 3). When VSMCs were pretreated with the specific ROCK inhibitor, Y27632, the expression level of differentiated proteins increased to nearly a normal level (0.76 ± 0.1-, 0.78 ± 0.15-fold of normal VSMCs for SM *α*-actin and calponin, respectively, *P* < 0.05). Overexpression of human DDAH2 in VSMCs also blunted the inhibiting effects of ADMA on differentiated marker protein expression in VSMCs.

### 3.4. Rho/ROCK Signal Transduction Pathway Induced by ADMA May Lead to the Activation of ERK Signal Pathway

**Figure 4:**
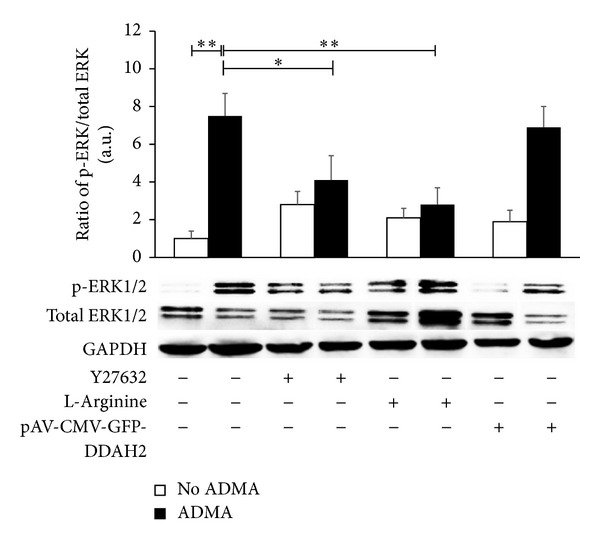
Rho/ROCK signal transduction pathway induced by ADMA leads to the activation of ERK signal pathway. ERK activity was determined after VSMCs were treated with or without ADMA for 15 min. The phosphorylation level of Tyr^202/204^-phosporylated ERK1/2 was detected. P-ERK1/2: phosphorous ERK1/2; ERK1/2: total ERK1/2. VSMCs pretreated by being transfected with pAV-CMV-GFP-DDAH2 or not, pretreated with or without Y27632 (10 *μ*M) for 30 min, and pretreated with or without L-arginine (1 mM) for two hours were indicated at the bottom. The graphs represent the relative expression level of every group by comparing to wild type VSMCs without ADMA treatment. Data shown are the mean ± SEM from three independent experiments, *P < 0.05;  **P < 0.01.

ERK and Rho/ROCK are indispensable for VSMC migration; therefore, we have investigated the possible signaling transduction and crosstalk between Rho/ROCK and ERK signaling. The results indicated that pretreatment of VSMCs with Y27632 (10 *μ*M) for 30 min significantly blocked the phosphorylation of ERK induced by ADMA for 15 min. L-Arginine significantly reduced ERK activation (Figure 4) but overexpression of DDAH2 in VSMCs did not exert significant effects on ERK activation. It is necessary to explore the different effects on ERK activation between pretreatment with L-arginine and overexpression of DDAH2 in VSMCs. Notwithstanding, these results suggest a unique signaling crosstalk between Rho/ROCK and ERK in mediating migration of VSMCs induced by ADMA.

### 3.5. ADMA Induces Morphological Changes of VSMCs

**Figure 5:**
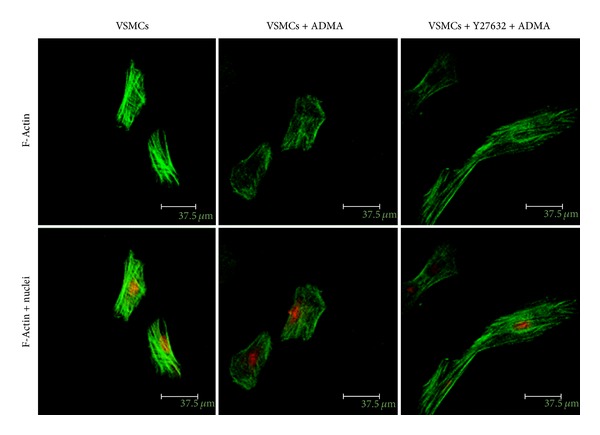
ADMA induces morphological changes of VSMCs. Confluent VSMCs (starved for 48 h in FCS-free DMEM without L-arginine) were pretreated with Y27632 (or not) and then incubated with ADMA or without ADMA at the concentration of 10 *μ*M for 12 h. F-Actin was stained with FITC-phalloidin and nucleolus was stained with PI.

Morphological changes of VSMCs are important hallmarks of the VSMCs differentiated state. As shown in Figure 5, immunofluorescence staining under confocal microscopy showed disassembly of actin fibres around the perinuclear region after treatment with ADMA for 12 h. In addition, VSMCs changing from a spindle-like to a polygonal shape was observed. VSMCs pretreated with Y27632 for 2 h readopted a spindle-like shape and reorganization of actin network from randomly oriented filaments into thicker and well-oriented fibres.

## 4. Discussion

The major findings presented in this study are that Rho/ROCK activation by ADMA mediates the ERK activation leading to migration of VSMCs and that Rho/ROCK activation by ADMA induces VSMCs phenotypic changes. This is in line with previous finding that ADMA activates Rho/ROCK in cardiac myocytes and endothelial cells. Thus, activation of Rho/ROCK represents one of the key signal transduction pathways originating from ADMA that mediates proatherogenic effects.

ADMA, the endogenous inhibitor of NOS, is synthesized during the methylation of protein arginine residues by protein arginine methyltransferases (PRMT) and is mainly metabolized by DDAH2 to citrulline and dimethylamine in cardiovascular system. More recently, it has been documented that ADMA is closely related to atherosclerosis in humans. In experimental animals, overexpression of ADMA causes atherosclerosis. Although the correlation between ADMA and atherosclerosis is well known, limited information was available previously regarding the mechanistic insights about ADMA and VSMCs migration, which is the critical process of plaque formation.

Up to date, many different intracellular signaling molecules have been implicated in cell migration, including Rho/ROCK, MAPK cascades. Our study demonstrates that ADMA can rapidly and significantly stimulate Rho/ROCK signal pathway that interpreted by the elevation of the affinity precipitation of Rho with GST-rhotekin fusion protein and by the enhancement of the phosphorylation of MYPT-1. Pretreatment with ROCK inhibitor, Y27632 completely reverses the induction of ADMA on ROCK, which is manifested by the reduction of phosphorylation level of MYPT-1. This is in line with previous observations that ADMA activates ROCK activity in endothelial cells [7]. Subsequent study suggests that VSMCs migration can be concomitantly induced by ADMA, which completely reversed by ROCK inhibitor Y27632. Thus, activation of Rho/ROCK represents one of the key signal transduction pathways that ADMA induces VSMCs migration.

MAPK, including ERK, c-Jun NH2-terminal kinase (JNK), and p38 MAP kinase (p38), plays the major role in stress-induced cellular responses, including cell proliferation, survival, or apoptosis [12, 13]. Recently, accumulating evidences have demonstrated that MAPK may be involved in VSMCs migration [14–17]. Zhan et al. have revealed that dominant-negative mutants of MAPK, including JNK, ERK, and p38, significantly decreased PDGF-BB–induced VSMC migration [17]. Ohtsu et al. also find the crosstalk between Rho/ROCK and JNK that mediates migration of VSMCs stimulated by angiotensin II [18]. In the present study, the phosphorylation of ERK is rapidly and significantly enhanced by ADMA treatment. In addition, when the Rho/ROCK signal pathway has been blocked by pretreatment with Y27632, the induction of ERK signal pathway by ADMA is completely abrogated. This suggests that the activation of Rho/ROCK signal transduction pathway induced by ADMA may lead to the activation of ERK signal pathway. This is in accordance with previous observations that Rho/ROCK pathway contributes to the activation of ERK pathway during myocardial cell hypertrophy [19].

There are two main ERK isoforms, ERK1 and ERK2, which share approximately 85% of amino acid identity, activated by the same stimuli, and believed to bear similar substrate recognition properties and subcellular localization [20]. ERK1 and ERK2 have been assumed to be functionally equivalent; recent studies have distinguished critical functional differences between these two proteins. In fact, significant differences between the two kinases also appear in the control of cell growth, in cultured fibroblasts, hepatocytes, and liver tumor. In previous studies, ADMA has been found to directly activate ERK1/2 in VSMC, but the difference has not been indicated. Here, we found the more phosphorylation level of ERK2 compared to ERK1, which indicates that ADMA selectively acts on the ERK2-induced signaling system in VSMC. There are probably many more direct and indirect mechanisms of ADMA-induced VSMC phenotype change via ERK2 that must be uncovered in future studies.

Both of DDAH2 and L-arginine block the effects of ADMA on the activation of Rho/ROCK and ERK as well as the induction of VSMCs migration in VSMCs. These results suggest that the activation of Rho/ROCK and ERK in VSMCs induced by ADMA may be related to the reduction of NO bioactivity. It is well known that ADMA, the endogenous inhibitor of NOS, is mainly metabolized by DDAH2 to citrulline and dimethylamine in the cardiovascular system [21]. Pharmacological inhibition of DDAH2 increases ADMA concentration and reduces NO production, whereas transgenic DDAH2 overexpression has the opposite effect both in vitro and in vivo [22]. In the present study, DDAH2 overexpression significantly attenuated the effects of ADMA on the activation of Rho/ROCK and ERK in VSMCs but failed to enhance SMC-specific marker expression. However, L-arginine completely reversed the effects of ADMA. Further investigations should be taken to distinguish this difference.

In fact, recently, an ApoE−/−/hDDAH1+/− transgenic mouse has been generated to overexpress the human isoform 1 of the ADMA-degrading enzyme dimethylarginine dimethylaminohydrolase (DDAH). They determined that overexpression of hDDAH1 reduced plaque formation in ApoE−/− mice by lowering ADMA, the first direct evidence of the role of ADMA in atherosclerotic plaque formation. Hence, although we did not find the evidence that ADMA induces atherosclerotic plaque formation via VSMC proliferation and migration, we can deduce that ADMA-caused VSMC proliferation and migration via RhoA/ROCK cell signaling transduction way may partly be attributed to atherosclerotic plaque formation.

Here, our present study indicates that ADMA induces the migration of VSMCs not only via the crosstalk between Rho/ROCK and ERK pathway, but also through the L-arginine/NO cascades. These findings are similar to the study of Suzuki et al., which have reported that the eNOS/NO cascade specifically targets the Rho/ROCK system to prevent vascular migration [23]. The exact functional consequence of the crosstalk between L-arginine/ADMA/NO and Rho/ROCK system may help to better understand the underlying mechanisms that ADMA induced VSMCs migration.

Apparently, the activation of Rho/ROCK in endothelial cells and VSMCs leads to opposite effect on cell migration. Maybe, the phenotype plasticity of VSMCs can help to distinguish this difference. It is well known that VSMCs retain phenotype plasticity, while endothelial cell does not. Phenotypic change is the prerequisite of migration in VSMCs. VSMCs phenotypic modulation is associated not only with change in the level of expression of differentiation proteins, but also with the reorganization of actin cytoskeleton [24]. The differentiated state of VSMCs is characterized by the elongated spindle shape of these cells and the assembly of actin cytoskeleton into parallel stress fibers. To ascertain whether or not ADMA inducing VSMCs migration via Rho/ROCK signal pathway is due to its effect on the VSMCs phenotype, cell morphology and actin cytoskeleton were examined.

In the present study, ADMA markedly inhibits the expression of *α*-actin and calponin. Disassembly and aggregation of actin skeleton around the perinuclear region after ADMA treatment are also presented. Pretreatment with Y27632 or L-arginine significantly enhanced the expression level of calponin and *α*-actin and completely abrogated the morphological changes of VSMCs induced by ADMA. This observation indicates that a phenotypic change in VSMCs induced by ADMA can be blocked by Rho/ROCK inhibitor Y27632 and by L-arginine. However, DDAH2 overexpression in VSMCs does not show significantly effects on phenotypic marker expression; it is not introduced in the morphological investigations.

This is an attractive finding because L-arginine is a semiessential amino acid that the dietetic application of L-arginine is the basic determinant of the L-arginine level in plasma, as the biosynthesis of L-arginine is not able to balance inadequate intake or deficiency. It is well known that most pharmaceuticals used to inhibit intimal hyperplasia are cytotoxic drugs. The long-term safety and efficacy of these drugs are still questionable while the semiessential amino acid L-arginine can both protect endothelial and maintain VSMCs differentiation phenotype. Findings of the present study suggest that L-arginine supplementation should be performed in individuals with the risk factors of cardiovascular disease or with high risk ratio of atherosclerosis or restenosis, either through arginine-rich foods or through health products.

Our findings presented here are limited within multipassaged cultured VSMCs. Future studies are necessary to confirm the presence of cascade in in vivo conditions or diseases associated with enhanced ADMA actions. The exact functional consequences of these crosstalks and their underlying mechanisms await further investigation.

In conclusion, ADMA activates Rho/ROCK activity, reduces SMC-specific markers expression, and induces VSMCs migration. The above-mentioned findings synergistically indicate that ADMA induces the phenotypic changes from a differentiated state to a dedifferentiated state that lead to the migration of VSMCs via the activation of Rho/ROCK signal transduction pathway. What is more, the semiessential amino acid L-arginine may reverse the detrimental effects on VSMCs migration and phenotypic changes induced by ADMA, which will contribute to the therapy and prevention of atherosclerosis.
